# Coupled Development of Salt Glands, Stomata, and Pavement Cells in *Limonium bicolor*

**DOI:** 10.3389/fpls.2021.745422

**Published:** 2021-12-09

**Authors:** Yaru Gao, Boqing Zhao, Xiangmei Jiao, Min Chen, Baoshan Wang, Fang Yuan

**Affiliations:** Shandong Provincial Key Laboratory of Plant Stress, College of Life Sciences, Shandong Normal University, Jinan, China

**Keywords:** leaf area, *Limonium bicolor*, pavement cell, phytohormone, salt gland, stomata

## Abstract

Salt-resistant plants have different mechanisms to limit the deleterious effects of high salt in soil; for example, recretohalophytes secrete salt from unique structures called salt glands. Salt glands are the first differentiated epidermal structure of the recretohalophyte sea lavender (*Limonium bicolor*), followed by stomata and pavement cells. While salt glands and stomata develop prior to leaf expansion, it is not clear whether these steps are connected. Here, we explored the effects of the five phytohormones salicylic acid, brassinolide, methyl jasmonate, gibberellic acid, and abscisic acid on the development of the first expanded leaf of *L. bicolor* and its potential connection to salt gland, stomata, and pavement cell differentiation. We calculated the total number of salt glands, stomata, and pavement cells, as well as leaf area and pavement cell area, and assessed the correlations between these parameters. We detected strong and positive correlations between salt gland number and pavement cell area, between stomatal number and pavement cell area, and between salt gland number and stomatal number. We observed evidence of coupling between the development of salt glands, stomata, and pavement cells in *L. bicolor*, which lays the foundation for further investigation of the mechanism behind salt gland development.

## Introduction

About 10% of arable land is affected by salinization worldwide, of which 2.5 × 10^9^ hm^2^ is irrigated (Ruan et al., 2010). This salinity severely affects plant growth and development and crop yields, especially for countries located in arid and semiarid climate zones (Rozema and Flowers, 2008). Saline land accounts for 4.9% (or 3.6 × 10^7^ hm^2^) of China’s arable land (Li J. et al., 2014). In recent years, the growth of the world population has placed great pressure on global food supply chains, including agricultural productivity. However, unreasonable irrigation and improper application of chemical fertilizers has resulted in secondary salinization of the soil, which is becoming increasingly problematic and in need of a long-term solution (Rengasamy, 2006). Approaches to fundamentally solve the problem of soil salinization have attracted much attention recently.

Most crops cannot survive or complete their life cycle when growing in saline soil (Fan, 2020), while halophytes, representing about 1% of the plant kingdom, can resistant high salt conditions and produce much biomass under appropriate NaCl concentrations (Flowers and Colmer, 2008). Halophytes are defined as plants that can survive and complete their life cycle at NaCl concentrations of 200 mM or above (Flowers and Colmer, 2008). Halophytes provide excellent models to study salt resistance, which could lead to new methods of mitigating soil salinization and increasing crop yield using transgenic techniques (Shabala, 2013; Yuan et al., 2016a).

Under salt stress conditions, halophytes adjust their physiology and biochemistry to reduce or alleviate the damage caused by high salinity (Hasanuzzaman et al., 2014). Halophytes can be classified into three categories based on their mechanism of resistance to high salinity: euhalophytes, pseudo-halophytes and recretohalophytes (Breckle, 1995). Recretohalophytes have unique salt secretory structures called salt glands, their most significant morphological and structural characteristics (Yuan et al., 2015). These salt glands can eliminate excessive salt ions from plant tissues, as seen in the recretohalophyte sea lavender (*Limonium bicolor*). Similarly, salt cedar (*Tamarix chinensis*) homogenizes salt concentrations between the surface and deeper layers of saline soil through salt glands (Feng et al., 2018). Sea milkwort (*Lysimachia maritima*, previously called *Glaux maritima* L.) also avoids high concentrations of Na^+^ and Cl^–^ ions in its tissues via salt glands (Rozema and Riphagen, 1977).

Given their importance in conferring salt resistance, the evolutionary origin and gene expression profile of salt glands have garnered substantial interest (Dassanayake and Larkin, 2017). Salt gland development has been studied in *Limonium vulgare* (Thomson, 1975). Salt glands arise from five consecutive cell divisions of a single epidermal cell to form a gland complex composed of 20 cells (Wiehe and Breckle, 1990). About 67 species distributed among 13 families possess salt glands (Yuan and Wang, 2020), but few have been investigated in details. *L. bicolor* is a typical recretohalophyte with salt glands on its leaf epidermis. It is considered a model halophyte to study salt gland development (Yuan et al., 2016a) due to the ease with which structures can be observed by autofluorescence and to a high-efficiency transformation system (Yuan et al., 2014). Each *L. bicolor* salt gland consists of 16 cells: four secretory cells, four accessory cells, four inner cup cells, and four outer cup cells (Yuan et al., 2016a). The continuous observation of leaf development revealed five typical developmental stages, starting with an undifferentiated stage, followed by the salt gland differentiation stage, stomatal differentiation stage, and pavement cell differentiation stage, and ending with the mature stage (Yuan et al., 2015).

Salt glands are therefore considered to be the first differentiated structure of the epidermis, and their numbers increase with leaf expansion (Leng et al., 2018), suggesting a possible relationship between salt glands and pavement cells along the expanding leaf. There is evidence that various treatments affect the number of salt glands and leaf area in a similar direction. For instance, calcium ions (Ca^2+^) (Ding et al., 2010), NaCl (Yuan et al., 2018) and exogenous nitric oxide (NO) (Ding, 2013) can promote the development of salt glands and increase total leaf area in *L. bicolor*. However, a systematic exploration of the possible relationship between salt glands, stomata, and pavement cells is lacking. Here, we applied five phytohormones known to influence salt gland development to investigate the potential links between epidermal cell types.

Phytohormones are small organic molecules that play a key role in plant metabolism, growth, and development at very low concentrations (Shi et al., 2017). When plants are subjected to various environmental stresses (such as salt stress), plant hormones also sustain continuous growth and development by balancing endogenous signals and exogenous stress (Yu et al., 2020). Methyl jasmonate (MeJA) can improve the resistance of *L. bicolor* to high salt conditions (Yuan et al., 2018). Melatonin has a similar effect by repressing the biosynthesis of abscisic acid (ABA) under high salinity, thus promoting seed germination and increasing the number of salt glands (Li et al., 2019, 2020). However, previous reports mainly employed exogenous treatment of large seedlings by spraying phytohormone solutions grown in soil or Hoagland’s nutrient solution, and none involved the direct addition of phytohormones to the growth medium to follow changes in leaf area or the number of salt glands, epidermal cells, or stomata. Here, five phytohormones [salicylic acid (SA), brassinolide (BL), MeJA, gibberellic acid (GA_3_) and ABA] were separately added in the media to investigate their effect on the differentiation and number of salt glands, stomata, and pavement cells from the first true expanded leaf to explore the possible relationships between salt glands, stomata, pavement cells, and leaf development.

## Materials and Methods

### Plant Materials

Seeds of *L. bicolor* were collected in October 2019 from the inland saline soil of the Yellow River Delta (N37°20′; E118°36′) in Dongying, Shandong, China. Uniform seeds were selected for sowing after storage at 4°C for 6 months.

### Phytohormone Treatments

The seeds of *L. bicolor* were initially surface-sterilized in 75% ethanol for 5 min, followed by soaking in 6% sodium hypochlorite solution with shaking for 15–20 min. Seeds were then washed using sterile water three to five times. The seeds were sown on growth medium containing various phytohormones and grown at 26/22°C (day/night) with a photoperiod of 16/8 h (day/night) and a light intensity of 600 μmol/m^2^/s.

All seeds were sown on Murashige and Skoog (MS) basal growth medium adjusted to pH 5.8–6.0 (Murashige and Skoog, 1962). The stock solutions of phytohormones were 50 mg/mL SA, 0.5 mg/mL BL, 10 μM MeJA, 0.1 mg/mL GA_3_, and 0.1 mg/mL ABA. MeJA was dissolved in absolute ethanol, while all other phytohormones were first dissolved in a small volume of absolute ethanol until full dissolution before the volume was adjusted with water. All stock solutions were filtered using a 0.2-μm filter.

Salicylic acid was added to MS medium to final concentrations of 25, 50, 75, 100, or 125 mg/L (Rajjou et al., 2006; Rivas-San Vicente and Plasencia, 2011). BL was added to MS medium at concentrations of 1.25, 2.5, 5, or 7.5 mg/L (Vogler et al., 2014). MeJA was added to a final concentration of 0.01 or 0.1 μM (Bazabakana et al., 1999; Chen et al., 2012). GA_3_ was added to a final concentration of 0.02, 0.04, 0.06, 0.08, or 0.1 mg/L (Li G. et al., 2014). ABA was added to a final concentration of 0.02, 0.04, 0.06, or 0.08 mg/L (Pan et al., 2020). Three replicates were sown for each concentration, and each replicate consisted of 20 seeds.

### Scoring the Numbers of Salt Glands, Stomata, and Pavement Cells, Leaf Area, and Pavement Cell Area

After growth for 15 days, the first true leaves were collected and fixed in a mixture of ethanol and acetic acid (3:1, v/v), then cleared in 70% ethanol, before being mounted in Hoyer’s solution (Meinke, and David, 1994). The leaves were observed by differential interference contrast (DIC) microscopy (ECLIPSE 80i, Nikon, Tokyo, Japan) with 330–380 nm ultraviolet (UV) excitation Images from five different fields were taken for each leaf, with 15 leaves per treatment. The average numbers of salt glands, pavement cells and stomata were calculated across the five fields using images acquired with a CCD camera (Nikon, Japan). Leaf area and pavement cell area were measured using ImageJ.

Salt gland density was calculated using the number of salt glands in a field, divided by the field area. The total number of salt glands was calculated as leaf area × salt gland density. Pavement cell density was calculated as the number of pavement cells in a field, divided by the field area, while the total number of epidermal cells was defined as leaf area × epidermal cell density. Stomatal density was calculated as the number of stomata in a field, divided by the field area, with the total number of stomata being leaf area × stomatal density.

In parallel, leaves were observed on an upright microscope (DM6B, Leica, Germany) to visualize pavement cells with a 19-mm field of view sCMOS camera with the LAS X Navigator Software.

### Data Analysis

SPSS (IBM, SPSS Statistics 25) was used for statistical analysis. Duncan’s multiple comparisons were performed to determine significant differences between samples, with a significance cutoff of *P* < 0.05. Analysis of variance (ANOVA) was used to determine statistical significance. All data collected from the five phytohormone treatments were normalized in SPSS to remove the influence of dimensions. These normalized values were saved as variables and used for correlation analysis according to the phytohormone. Statistical significance was determined by two-tailed Student’s *t*-test for *P* < 0.05 and *P* < 0.01.

## Results

### The Development of Salt Glands Is Promoted Below 25 mg/L Salicylic Acid

**FIGURE 1:**
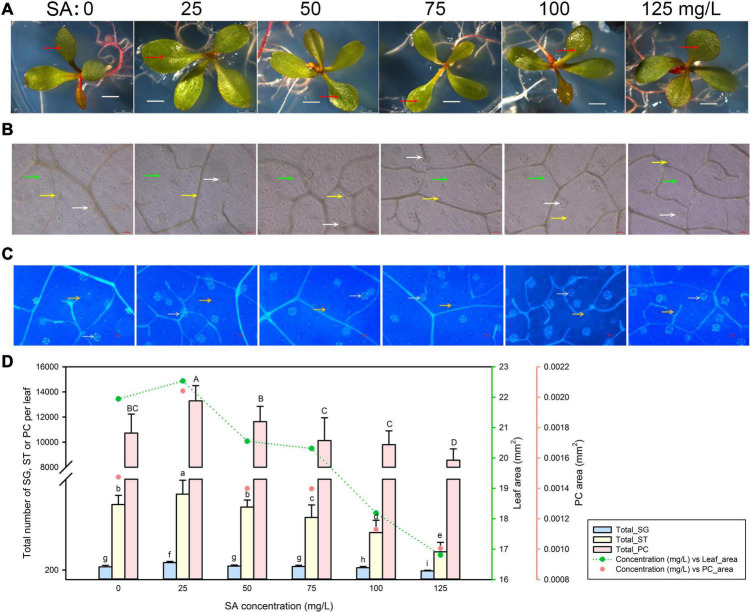
Representative phenotypes of *Limonium bicolor* seedlings exposed to various concentrations of salicylic acid (SA) for 15 days. **(A)** Phenotype of *L. bicolor* treated with different concentrations of SA for 15 days. Red arrows indicate the first expanded true leaf. **(B)** Representative leaf surface images using an upright microscope. Bar = 25 μm. White arrows, salt glands; yellow arrows, stomata; green arrows, pavement cells. **(C)** Visualization of salt glands and stomata by autofluorescence under ultraviolet light. Bar = 25 μm. White arrows, salt glands; yellow arrows, stomata. **(D)** Mean numbers of salt glands, stomata, and pavement cells, and total area of expanded leaf and pavement cells as a function of SA concentration. Green line, leaf area; red circles, pavement cell area. Data are shown as means ± SD (*n* = 15). Different letters indicate significant differences at *P* < 0.05 using Duncan’s multiple test. SG, salt gland; ST, stoma; PC, pavement cell.

We first germinated *L. bicolor* seeds on MS medium containing a range of SA concentrations (0–125 mg/L) to investigate the development of salt glands, stomata, and pavement cells from the fully expanded first true leaf (Figure 1A). We measured the area of the epidermis occupied by pavement cells with an upright microscope (Figure 1B) and scored the number of salt glands and stomata using autofluorescence resulting from UV excitation between 330 and 380 nm (Figure 1C). Given that salt gland density varied under different treatments (Supplementary Figure 1) due to the different leaf area, the total salt gland number on the first true leaf are further used to be compared with stomata and pavement cells.

The area of the first true leaf increased slightly when treated with 25 mg/L SA relative to control leaves but then decreased with higher SA concentrations (>25 mg/L) (Figure 1D). We observed the same trend for the leaf area covered by pavement cells, which was highest at 25 mg/L SA. Likewise, the number of salt glands, stomata, and pavement cells followed the same pattern, with the greatest cell numbers obtained with 25 mg/L SA, and fewer cells at higher SA concentrations (>25 mg/L) (Figure 1D).

### Leaf Development of *Limonium bicolor* Is Promoted at 1.25 mg/L Brassinolide

**FIGURE 2:**
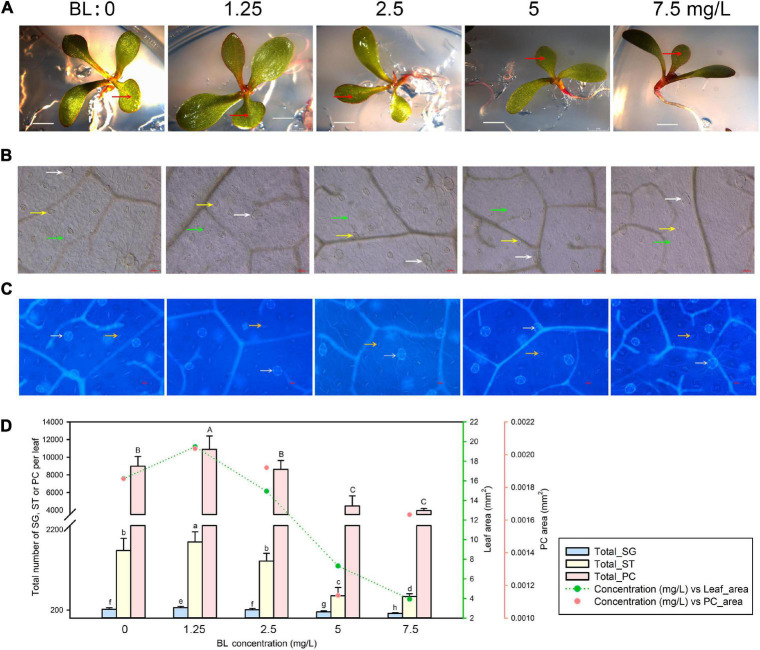
Representative phenotypes of *L. bicolor* seedlings exposed to various concentrations of brassinolide (BL) for 15 days. **(A)** Phenotype of *L. bicolor* treated with different concentrations of BL for 15 days. Red arrows indicate the first expanded true leaf. **(B)** Representative leaf surface images using an upright microscope. Bar = 25 μm. White arrows, salt glands; yellow arrows, stomata; green arrows, pavement cells. **(C)** Visualization of salt glands and stomata by autofluorescence under ultraviolet light. Bar = 25 μm. White arrows, salt glands; yellow arrows, stomata. **(D)** Mean numbers of salt glands, stomata, and pavement cells, and total area of expanded leaf and pavement cells as a function of BL concentration. Green line, leaf area; red circles, pavement cell area. Data are shown as means ± SD (*n* = 15). Different letters indicate significant differences at *P* < 0.05 using Duncan’s multiple test. SG, salt gland; ST, stoma; PC, pavement cell.

We next germinated *L. bicolor* seeds and followed seedling growth on MS medium containing 0–7.5 mg/L BL (Figure 2A). As with SA, we observed pavement cells (Figure 2B), salt glands, and stomata (Figure 2C). Again, leaf area and pavement cell area reached their highest values at the lowest phytohormone concentration (here 1.25 mg/L BL) (Figure 2D), both exhibiting a gradual decrease at higher concentrations (>1.25 mg/L) of BL. The total numbers of salt glands, stomata, and pavement cells followed similar trends with BL concentration (Figure 2D), peaking at 1.25 mg/L BL, before dropping with higher BL concentrations (>1.25 mg/L).

### Methyl Jasmonate Inhibits Leaf Development in *Limonium bicolor*

**FIGURE 3:**
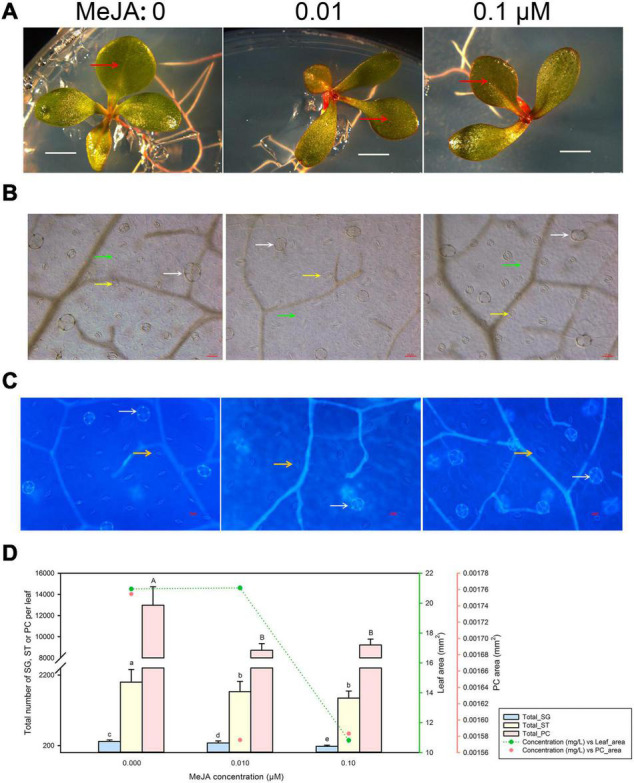
Representative phenotypes of *L. bicolor* seedlings exposed to various concentrations of methyl jasmonate (MeJA) for 15 days. **(A)** Phenotype of *L. bicolor* treated with different concentrations of MeJA for 15 days. Red arrows indicate the first expanded true leaf. **(B)** Representative leaf surface images using an upright microscope. Bar = 25 μm. White arrows, salt glands; yellow arrows, stomata; green arrows, pavement cells. **(C)** Visualization of salt glands and stomata by autofluorescence under ultraviolet light. Bar = 25 μm. White arrows, salt glands; yellow arrows, stomata. **(D)** Mean numbers of salt glands, stomata, and pavement cells, and total area of expanded leaf and pavement cells as a function of MeJA concentration. Green line, leaf area; red circles, pavement cell area. Data are shown as means ± SD (*n* = 15). Different letters indicate significant differences at *P* < 0.05 using Duncan’s multiple test. SG, salt gland; ST, stoma; PC, pavement cell.

Minute amounts of MeJA can substantially affect leaf development, as evidenced by the inhibition of seedling growth we observed with 0.1 μM MeJA (Figure 3A). Accordingly, we determined the developmental status of salt glands, stomata, and pavement cells at low MeJA concentrations of 0.01 and 0.1 μM (Figures 3B,C). Leaf area was markedly smaller upon treatment with 0.1 μM MeJA but not with 0.01 μM MeJA, while pavement cell area already diminished at MeJA concentrations as low as 0.01 μM compared to control seedlings (Figure 3D). Similarly, increasing MeJA concentrations were accompanied by a reduction in the total numbers of salt glands, stomata, and pavement cells relative to control conditions.

### Gibberellic Acid Treatment Inhibits Leaf Development in *Limonium bicolor*

**FIGURE 4:**
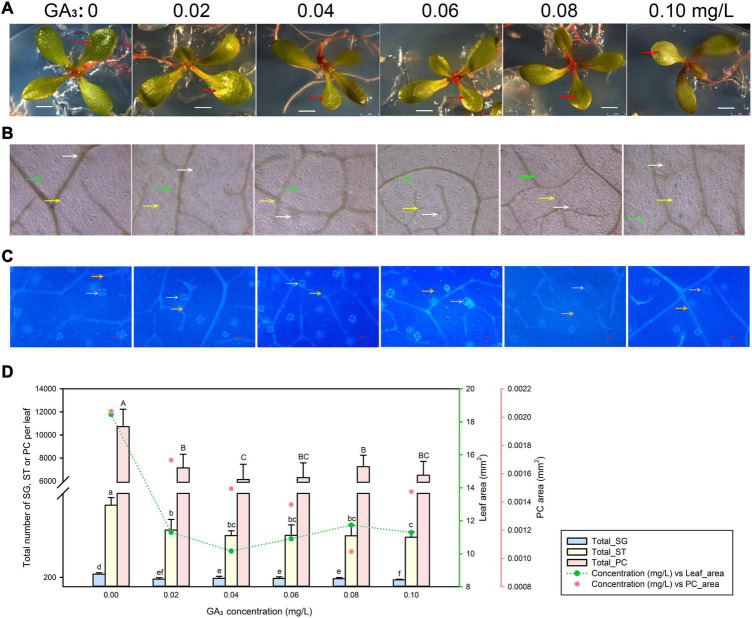
Representative phenotypes of *L. bicolor* seedlings exposed to various concentrations of gibberellic acid (GA_3_) for 15 days. **(A)** Phenotype of *L. bicolor* treated with different concentrations of GA_3_ for 15 days. Red arrows indicate the first expanded true leaf. **(B)** Representative leaf surface images using an upright microscope. Bar = 25 μm. White arrows, salt glands; yellow arrows, stomata; green arrows, pavement cells. **(C)** Visualization of salt glands and stomata by autofluorescence under ultraviolet light. Bar = 25 μm. White arrows, salt glands; yellow arrows, stomata. **(D)** Mean numbers of salt glands, stomata, and pavement cells, and total area of expanded leaf and pavement cells as a function of GA_3_ concentration. Green line, leaf area; red circles, pavement cell area. Data are shown as means ± SD (*n* = 15). Different letters indicate significant differences at *P* < 0.05 using Duncan’s multiple test. SG, salt gland; ST, stoma; PC, pavement cell.

We exposed seeds to five different GA_3_ concentrations ranging from 0.02 to 0.1 mg/L (along with a control not treated with GA_3_) and characterized leaf development (Figure 4A), pavement cell expansion (Figure 4B), and the numbers of salt glands and stomata (Figure 4C). Even the lowest GA_3_ concentration of 0.02 mg/L limited leaf expansion; raising GA_3_ concentrations to 0.1 mg/L did not substantially further reduce leaf area. Pavement cell area showed a similar trend (Figure 4D). Likewise, the numbers of salt glands, stomata, and pavement cells dropped with 0.02 mg/L GA_3_, but then remained constant with higher GA_3_ concentrations (>0.02 mg/L) (Figure 4D).

### Leaf Development of *Limonium bicolor* Is Suppressed by Abscisic Acid

**FIGURE 5:**
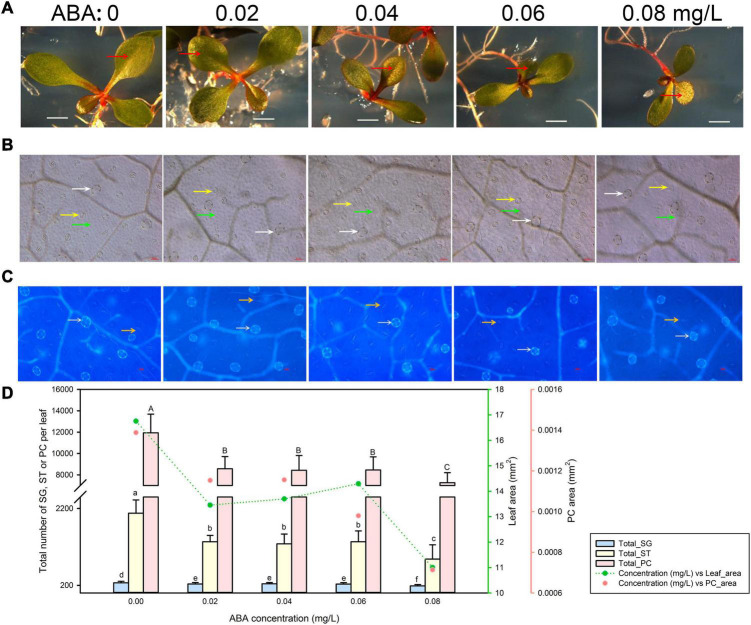
Representative phenotypes of *L. bicolor* seedlings exposed to various concentrations of abscisic acid (ABA) for 15 days. **(A)** Phenotype of *L. bicolor* treated with different concentrations of ABA for 15 days. Red arrows indicate the first expanded true leaf. **(B)** Representative leaf surface images using an upright microscope. Bar = 25 μm. White arrows, salt glands; yellow arrows, stomata; green arrows, pavement cells. **(C)** Visualization of salt glands and stomata by autofluorescence under ultraviolet light. Bar = 25 μm. White arrows, salt glands; yellow arrows, stomata. **(D)** Mean number of salt glands, stomata, and pavement cells, and total area of expanded leaf and pavement cells as a function of ABA concentration. Green line, leaf area; red circles, pavement cell area. Data are shown as means ± SD (*n* = 15). Different letters indicate significant differences at *P* < 0.05 using Duncan’s multiple test. SG, salt gland; ST, stoma; PC, pavement cell.

Finally, we tested the effects of ABA treatment on leaf growth (Figure 5A) and epidermis development (Figures 5B,C). Low and intermediate ABA concentrations (0.02–0.06 mg/L) reduced leaf and pavement cell areas to roughly the same extent (Figure 5D). However, the highest ABA concentration applied (0.08 mg/L) further reduced both leaf and pavement cell areas. The total numbers of salt glands, stomata, and pavement cells displayed the same trends as leaf area (Figure 5D).

### Correlation Between Total Numbers of Salt Glands, Stomata, and Pavement Cells; Leaf Area; and Pavement Cell Area Under Different Treatments

We then explored the correlations between the total numbers of salt glands, stomata, and pavement cells; leaf area; and pavement cell area for each phytohormone using SPSS. For SA treatments, the total salt gland number showed a significant (*P* = 0.01) and positive correlation with leaf area, pavement cell area, total stomatal number, and pavement cell number (Supplementary Table 1). We also obtained strong, positive correlations between stomatal number and leaf area, as well as stomatal number and pavement cell number, indicating that the development of salt glands, stomata, and pavement cells might be coupled or coordinated during leaf expansion.

We then applied the same analysis to the other four phytohormones (Supplementary Tables 2–5). We observed similar results, supporting the notion that the number of salt glands is highly correlated with all other parameters tested. We therefore conclude that larger leaves will present more salt glands, stomata, and pavement cells.

Finally, we integrated the data from all five phytohormones after normalization to remove any scaling effects and repeated the correlation analysis between the total numbers of salt glands, stomata, and pavement cells; leaf area; and pavement cell area (Table 1). Again, we obtained highly significant and positive correlations between salt glands and other parameters.

**TABLE 1:** Correlation analysis between total salt glands (Total_SG) and the other four parameters: total stomata (Total_ST), total pavement cells (Total_PC), leaf area (Leaf_area), and pavement cell area (PC_Area) upon treatment with the five phytohormones salicylic acid (SA), brassinolide (BL), methyl jasmonate (MeJA), gibberellic acid (GA), and abscisic acid (ABA) using Pearson’s correlation analysis.

	Mean	Std. D	Total_SG	Total_ST	Total_PC	Leaf_Area	PC_Area
**Correlations**							
Total_SG	2.317E + 02	6.780E + 01	1.000				
Total_ST	1.348E + 03	4.930E + 02	0.515**	1.000			
Total_PC	9.001E + 03	2.670E + 03	0.609**	0.634**	1.000		
Leaf_Area	1.508E + 01	4.934E + 00	0.682**	0.503**	0.655**	1.000	
PC_Area	1.483E-03	4.321E-04	0.264**	0.419**	0.285**	0.189**	1.000

**Correlation is significant at the 0.01 level (2-tailed).

## Discussion

Leaf development is controlled by a complex regulatory network. Salt glands are a specific derived epidermal structure of recretohalophytes, but how the salt gland differentiate from single epidermal cell is still unclear. Here, we conducted visual assessments and quantifications of the numbers of salt glands, stomata, and pavement cells as well as leaf and pavement cell areas in seedlings exposed to five phytohormones to explore possible relationships during leaf development. All parameters were strongly and positively correlated for all phytohormones tested.

Salicylic acid is an endogenous defense phytohormone that regulates many aspects of plant growth and development, especially during stress responses (Klessig et al., 2018) by reducing the accumulation of reactive oxygen species (ROS; Ma et al., 2017). Exogenous SA treatment can promote germination of Arabidopsis (*Arabidopsis thaliana*) seeds exposed to high salinity (Lee et al., 2010). Here, in *L. bicolor*, we added SA to the growth medium to observe its effects on the development of salt glands, stomata, and pavement cells.

When plants are exposed to salt stress, brassinosteroids can result in hormonal stress dose–dependent biphasic effects. Increasing BL levels or enhancing BL signals can increase plant resistance to salt (Liu et al., 2020). Treatment with exogenous BL improves the salt tolerance of perennial ryegrass (*Lolium perenne* L.) by increasing the activity of antioxidant enzymes and proline content (Wu et al., 2017). Similar to previous studies (Asami et al., 2005), low concentrations of BL (1.25 mg/L) promoted leaf development in *L. bicolor* in this study, as well as development of salt glands, stomata, and pavement cells. With higher BL concentrations (>2.5 mg/L), we observed a typical inhibition response for leaf development, which is also consistent with a previous report that high BL concentrations inhibit the seedling development in Arabidopsis (Bao et al., 2004).

The phytohormones MeJA, GA_3_, and ABA limited leaf development and salt gland differentiation (Yuan et al., 2016b,^2018^). As a key phytohormone in plant defenses against pathogenic microorganisms and pests (Yuan et al., 2018), JA increases the transcription of genes encoding antioxidant enzymes in wheat (*Triticum aestivum*) seedlings, thereby improving their tolerance to salt stress (Qiu et al., 2014). However, in the current results, MeJA added to the growth medium inhibited leaf development of *L. bicolor*. We obtained similar inhibitory effects with GA_3_ treatments. GA_3_ is a growth-promoting plant hormone (Verma et al., 2016) that relieves seed dormancy, promotes stem elongation (Daviere and Achard, 2013), and reduces ROS contents during abiotic stress conditions (Colebrook et al., 2014). A rare role was also reported for GA_3_ in the development of epidermal structures. Low GA_3_ concentrations (0.02 mg/L) inhibited leaf development and the expansion of all leaf structures. Similar results were seen in seedlings treated with the stress phytohormone ABA, which responds to salt stress by regulating stomatal movements, increasing intracellular Ca^2+^ concentrations, and increasing ROS levels (Yu et al., 2020). In Arabidopsis, ABA sensitivity is typically reduced to improve tolerance to abiotic stress (Xu et al., 2020). We noted the repression of salt gland and stomatal development upon exposure to ABA. While ABA can induce stomatal closure (Postiglione and Muday, 2020), our present results reveal for the first time a relationship between stomatal development and ABA treatment.

### Total Salt Gland Number Versus Pavement Cell Number

As different phytohormones have different effects on leaf development, we combined all normalized data to determine the overall correlation between leaf development parameters. We detected strong correlations between salt gland number and pavement cell area, and between salt gland number and leaf area when considering each phytohormone separately (Supplementary Tables 1–5) or in combination (Table 1). In previous studies of *L. bicolor*, salt gland number always showed a positive correlation with leaf area (Yuan et al., 2018); for example, Ca^2+^ treatment enhanced salt gland development and leaf expansion (Ding et al., 2010). Here, we observed strong and positive correlations between salt gland number and leaf area, indicating that salt gland development is coupled with leaf development.

### Total Salt Gland Number Versus Stomata Number

Salt glands are not the only epidermal structure of *L. bicolor*. Stomata also responded to the different phytohormone treatments. The development of salt glands and stomata is thought to take place at distinct leaf developmental stages (Yuan et al., 2015). We obtained very strong, positive correlations between the numbers of salt glands and stomata, indicative of an indirect connection between salt gland and stomatal development. Moreover, we noted a positive correlation between stomatal number and pavement cell area, which was consistent with a previous study reporting that stomatal development is correlated with leaf area (Gay and Hurd, 2010).

Besides of the relationship between salt gland and pavement cell or stomata, there is an interesting phenomenon that no trichome distributed on the surface of *L. bicolor*. Given that trichomes also have the similar distribution pattern to salt gland, and trichomes from some species are reported to have secretory function (Wagner et al., 2004; Olsson et al., 2009), it is believed that there may be some homologous relationship between salt gland and trichome (Yuan et al., 2015). More will be verified by transformation the homologous genes involved in trichome development and salt gland development, such as a WD40-repeat protein (Yuan et al., 2019) and MYB transcription factor LbTRY (Leng et al., 2021).

**FIGURE 6:**
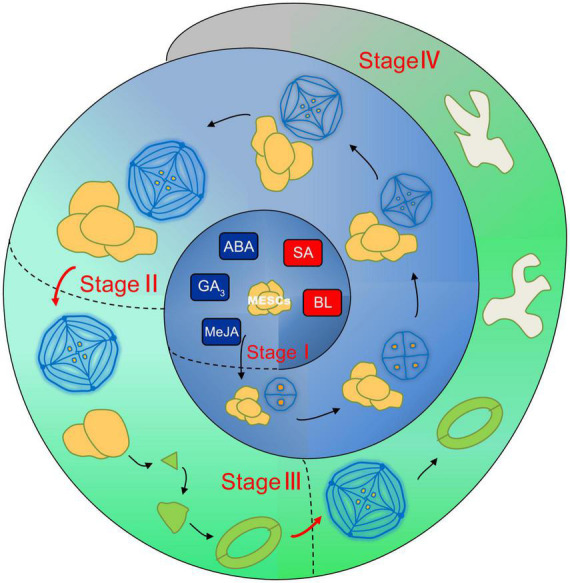
The schematic model of coupled leaf development in *L. bicolor*. Four distinct stages are divided including stage I (MESCs undifferentiation stage), stage II (from MESCs to salt gland), stage III (from MESCs to stomata) and stage IV (from MESCs to pavement cell). In stage I, SA and BL promote the further differentiation of MESCs, while GA_3_, MeJA and ABA inhibit this process. In stage II, salt glands are formed through two-cell, four-cell, eight-cell, twelve-cell and sixteen-cell. Stomata emerged in stage III and pavement cells formed in stage IV.

Based on the former proposed salt gland development pattern (Yuan et al., 2015; Xu et al., 2020), we further improve the leaf development model (Figure 6) including MESCs (multipotent epidermal stem cells) stage, salt gland differentiation stage, stomata stage and pavement cell formation stage. The plant regulators proposed in the current report may directly act on the regulation of MESCs with promotion of SA and BL, and inhibition of GA_3_, MeJA and ABA to further differentiate into different cell types. In conclusion, the leaf area of expanded leaves is a good direct indicator of the number of salt glands, stomata, and pavement cells. Salt glands on *L. bicolor* leaves can excrete excess Na^+^ out of the plant to avoid salt overaccumulation, but their development is not disconnected from that of the rest of the leaf. Our present results revealed connections between the development of salt glands, stomata, and pavement cells, which will be benefit to the further study of salt glands development.

## Data Availability Statement

The raw data supporting the conclusions of this article will be made available by the authors, without undue reservation.

## Author Contributions

FY and MC designed the research. YG, BZ, and XJ performed the research. YG analyzed the data. YG and FY wrote the manuscript. FY and BW revised the manuscript. All authors contributed to the article and approved the submitted version.

## Conflict of Interest

The authors declare that the research was conducted in the absence of any commercial or financial relationships that could be construed as a potential conflict of interest.

## Publisher’s Note

All claims expressed in this article are solely those of the authors and do not necessarily represent those of their affiliated organizations, or those of the publisher, the editors and the reviewers. Any product that may be evaluated in this article, or claim that may be made by its manufacturer, is not guaranteed or endorsed by the publisher.

## Supplementary Material

Supplementary Figure 1 Salt gland density of the first true leaf under different hormone treatments.

## References

[B1] Asami T. Nakano T. Fujioka S. (2005). Plant brassinosteroid hormones. *Vitam Horm* 72 479–504. 10.1016/S0083-6729(05)72014-8 16492480

[B2] Bao F. Shen J. Brady S. R. Muday G. K. Asami T. Yang Z. (2004). Brassinosteroids Interact with Auxin to Promote Lateral Root Development in Arabidopsis. *Plant Physiol.* 134:1624. 10.1104/pp.103.036897 15047895 PMC419836

[B3] Bazabakana R. Fauconnier M.-L. Diallo B. Dupont J. Homes J. El Jaziri M. (1999). Control of *Dioscorea alata* microtuber dormancy and germination by jasmonic acid. *Plant Growth Regulat.* 27 113–117. 10.1023/A:1006199432339

[B4] Breckle S.-W. (1995). How do halophytes overcome salinity? *Biol. Salt Toler. Plants* 1995 199–213.

[B5] Chen R. Jiang H. Li L. Zhai Q. Qi L. Zhou W. (2012). The Arabidopsis mediator subunit MED25 differentially regulates jasmonate and abscisic acid signaling through interacting with the MYC2 and ABI5 transcription factors. *Plant Cell* 24 2898–2916. 10.1105/tpc.112.098277 22822206 PMC3426122

[B6] Colebrook E. H. Thomas S. G. Phillips A. L. Hedden P. (2014). The role of gibberellin signalling in plant responses to abiotic stress. *J. Exp. Biol.* 217 67–75. 10.1242/jeb.089938 24353205

[B7] Dassanayake M. Larkin J. C. (2017). Making Plants Break a Sweat: the Structure. Function, and Evolution of Plant Salt Glands. *Front. Plant Sci.* 8:406. 10.3389/fpls.2017.00724 28400779 PMC5368257

[B8] Daviere J. M. Achard P. (2013). Gibberellin signaling in plants. *Development* 140 1147–1151. 10.1242/dev.087650 23444347

[B9] Ding F. (2013). Effects of salinity and nitric oxide donor sodium nitroprusside (SNP) on development and salt secretion of salt glands of Limonium bicolor. *Acta Physiol. Plant.* 35 741–747. 10.1007/s11738-012-1114-8

[B10] Ding F. Chen M. Sui N. Wang B. S. (2010). Ca2+ significantly enhanced development and salt-secretion rate of salt glands of Limonium bicolor under NaCl treatment. *South Afr. J. Bot.* 76 95–101. 10.1016/j.sajb.2009.09.001

[B11] Fan C. (2020). Genetic mechanisms of salt stress responses in halophytes. *Plant Signal Behav.* 15:1704528. 10.1080/15592324.2019.1704528 31868075 PMC7012083

[B12] Feng X. An P. Li X. Guo K. Yang C. Liu X. (2018). Spatiotemporal heterogeneity of soil water and salinity after establishment of dense-foliage Tamarix chinensis on coastal saline land. *Ecolog. Eng.* 121 104–113. 10.1016/j.ecoleng.2017.06.031

[B13] Flowers T. J. Colmer T. D. (2008). Salinity tolerance in halophytes. *New Phytol.* 179 945–963. 10.1111/j.1469-8137.2008.02531.x 18565144

[B14] Gay A. P. Hurd R. G. J. N. P. (2010). The influence of light on stomatal density in the tomato. *New Phytol.* 75 37–46. 10.1111/j.1469-8137.1975.tb01368.x

[B15] Hasanuzzaman M. Nahar K. Alam M. M. Bhowmik P. C. Hossain M. A. Rahman M. M. (2014). Potential Use of Halophytes to Remediate Saline Soils. *BioMed. Res. Internat.* 2014 1–12. 10.1155/2014/589341 25110683 PMC4109415

[B16] Klessig D. F. Choi H. W. Dempsey D. A. (2018). Systemic Acquired Resistance and Salicylic Acid: Past, Present, and Future. *Mol. Plant Microbe Interact.* 31 871–888. 10.1094/MPMI-03-18-0067-CR 29781762

[B17] Lee S. Kim S. G. Park C. M. (2010). Salicylic acid promotes seed germination under high salinity by modulating antioxidant activity in Arabidopsis. *New Phytol.* 188 626–637. 10.1111/j.1469-8137.2010.03378.x 20663063

[B18] Leng B. Y. Yuan F. Dong X. X. Wang B. S. (2018). Salt gland distribution in limonium bicolor at the individual level. *IOP Conference Series: Earth and Environmental Science* 113:012202 10.1088/1755-1315/113/1/012202

[B19] Leng B. Wang X. Yuan F. Zhang H. Lu C. Chen M. (2021). Heterologous expression of the *Limonium bicolor* MYB transcription factor LbTRY in *Arabidopsis thaliana* increases salt sensitivity by modifying root hair development and osmotic homeostasis. *Plant Sci.* 302:110704. 10.1016/j.plantsci.2020.110704 33288017

[B20] Li G. Zhu C. Gan L. Ng D. Xia K. (2014). GA3 enhances root responsiveness to exogenous IAA by modulating auxin transport and signalling in Arabidopsis. *Plant Cell Rep.* 34 483–494. 10.1007/s00299-014-1728-y 25540118

[B21] Li J. Pu L. Han M. Zhu M. Zhang R. Xiang Y. (2014). Soil salinization research in China: Advances and prospects. *J. Geograph. Sci.* 24 943–960. 10.1007/s11442-014-1130-2

[B22] Li J. Yuan F. Liu Y. Zhang M. Liu Y. Zhao Y. (2020). Exogenous melatonin enhances salt secretion from salt glands by upregulating the expression of ion transporter and vesicle transport genes in Limonium bicolor. *BMC Plant Biol.* 20:493. 10.1186/s12870-020-02703-x 33109099 PMC7590734

[B23] Li J. Zhao C. Zhang M. Yuan F. Chen M. (2019). Exogenous melatonin improves seed germination in Limonium bicolor under salt stress. *Plant Signal Behav.* 14:1659705. 10.1080/15592324.2019.1659705 31460852 PMC6804724

[B24] Liu J. Yang R. Jian N. Wei L. Ye L. Wang R. (2020). Putrescine metabolism modulates the biphasic effects of brassinosteroids on canola and Arabidopsis salt tolerance. *Plant Cell Environ.* 43 1348–1359. 10.1111/pce.13757 32176351

[B25] Ma X. Zheng J. Zhang X. Hu Q. Qian R. (2017). Salicylic Acid Alleviates the Adverse Effects of Salt Stress on Dianthus superbus (Caryophyllaceae) by Activating Photosynthesis, Protecting Morphological Structure, and Enhancing the Antioxidant System. *Front. Plant Sci.* 8:600. 10.3389/fpls.2017.00600 28484476 PMC5399920

[B26] Meinke David W. (1994). 10 Seed Development in Arabidopsis thaliana. *Cold Spring Harbor Monograph Archive* 1994:27.

[B27] Murashige T. Skoog F. (1962). A revised medium for rapid growth and bioassays with tobacco tissue cultures. *Physiol. Plant.* 15, 473–497.

[B28] Olsson M. E. Olofsson L. M. Lindahl A. L. Lundgren A. Brodelius M. Brodelius P. E. (2009). Localization of enzymes of artemisinin biosynthesis to the apical cells of glandular secretory trichomes of *Artemisia annua* L. *Phytochemistry* 70 1123–1128. 10.1016/j.phytochem.2009.07.009 19664791

[B29] Pan W. You Y. Shentu J. L. Weng Y. N. Wang S. T. Xu Q. R. (2020). Abscisic acid (ABA)-importing transporter 1 (AIT1) contributes to the inhibition of Cd accumulation via exogenous ABA application in Arabidopsis. *J. Hazard Mater.* 391:122189. 10.1016/j.jhazmat.2020.122189 32044630

[B30] Postiglione A. E. Muday G. K. (2020). The Role of ROS Homeostasis in ABA-Induced Guard Cell Signaling. *Front. Plant Sci.* 11:968. 10.3389/fpls.2020.00968 32695131 PMC7338657

[B31] Qiu Z. Guo J. Zhu A. Zhang L. Zhang M. (2014). Exogenous jasmonic acid can enhance tolerance of wheat seedlings to salt stress. *Ecotoxicol. Environ. Saf.* 104 202–208. 10.1016/j.ecoenv.2014.03.014 24726929

[B32] Rajjou L. Belghazi M. Huguet R. Robin C. Moreau A. Job C. (2006). Proteomic Investigation of the Effect of Salicylic Acid on Arabidopsis Seed Germination and Establishment of Early Defense Mechanisms. *Plant Physiol.* 141 910–923. 10.1104/pp.106.082057 16679420 PMC1489900

[B33] Rengasamy P. (2006). World salinization with emphasis on Australia. *J. Exp. Bot.* 57 1017–1023. 10.1093/jxb/erj108 16510516

[B34] Rivas-San Vicente M. Plasencia J. (2011). Salicylic acid beyond defence: its role in plant growth and development. *J. Exp. Bot.* 62 3321–3338. 10.1093/jxb/err031 21357767

[B35] Rozema J. Flowers T. (2008). Ecology. Crops for a salinized world. *Science* 322 1478–1480. 10.1126/science.1168572 19056965

[B36] Rozema J. Riphagen I. (1977). Physiology and ecologic relevance of salt secretion by the salt gland of Glaux maritima L. *Oecologia* 29 349–357. 10.1007/BF00345808 28309095

[B37] Ruan C.-J. Da Silva J. A. T. Mopper S. Qin P. Lutts S. (2010). Halophyte Improvement for a Salinized World. *Crit. Rev. Plant Sci.* 29 329–359. 10.1080/07352689.2010.524517

[B38] Shabala S. (2013). Learning from halophytes: physiological basis and strategies to improve abiotic stress tolerance in crops. *Ann. Bot.* 112 1209–1221. 10.1093/aob/mct205 24085482 PMC3806534

[B39] Shi T. Q. Peng H. Zeng S. Y. Ji R. Y. Shi K. Huang H. (2017). Microbial production of plant hormones: Opportunities and challenges. *Bioengineered* 8 124–128. 10.1080/21655979.2016.1212138 27459344 PMC5398602

[B40] Thomson W. W. (1975). “The structure and function of salt glands,” in *Plants Saline Environments*, Vol. 15, eds Poljakoff-Mayber A. Gale J. (Berlin: Springer), 118–146.

[B41] Verma V. Ravindran P. Kumar P. P. (2016). Plant hormone-mediated regulation of stress responses. *BMC Plant Biol.* 16:86. 10.1186/s12870-016-0771-y 27079791 PMC4831116

[B42] Vogler F. Schmalzl C. Englhart M. Bircheneder M. Sprunck S. (2014). Brassinosteroids promote Arabidopsis pollen germination and growth. *Plant Reprod* 27 153–167. 10.1007/s00497-014-0247-x 25077683

[B43] Wagner G. J. Wang E. Shepherd R. W. (2004). New Approaches for Studying and Exploiting an Old Protuberance, the Plant Trichome. *Ann. Bot.* 93 3–11. 10.1093/aob/mch011 14678941 PMC4242265

[B44] Wiehe W. Breckle S. W. (1990). Die Ontogenese der Salzdrüsen von Limonium (Plumbaginaceae); The Ontogenesis of the Salt Glands of Limonium (Plumbaginaceae). *Botanica Acta* 103 107–110. 10.1111/j.1438-8677.1990.tb00135.x

[B45] Wu W. Zhang Q. Ervin E. H. Yang Z. Zhang X. (2017). Physiological Mechanism of Enhancing Salt Stress Tolerance of Perennial Ryegrass by 24-Epibrassinolide. *Front. Plant Sci.* 2017:8. 10.3389/fpls.2017.01017 28674542 PMC5474491

[B46] Xu Y. Jiao X. Wang X. Zhang H. Wang B. Yuan F. (2020). Importin-β From the Recretohalophyte Limonium bicolor Enhances Salt Tolerance in Arabidopsis thaliana by Reducing Root Hair Development and Abscisic Acid Sensitivity. *Front. Plant Sci.* 11:582459. 10.3389/fpls.2020.582459 33519843 PMC7838111

[B47] Yu Z. Duan X. Luo L. Dai S. Ding Z. Xia G. (2020). How Plant Hormones Mediate Salt Stress Responses. *Trends Plant Sci.* 25 1117–1130. 10.1016/j.tplants.2020.06.008 32675014

[B48] Yuan F. Wang B. (2020). Adaptation of Recretohalophytes to Salinity. *Handbook Halophytes* 2020 1–21. 10.1007/978-3-030-17854-3_32-1

[B49] Yuan F. Chen M. Yang J. Leng B. Wang B. (2014). A system for the transformation and regeneration of the recretohalophyte Limonium bicolor. *Vitro Cell. Dev. Biol. Plant* 50 610–617. 10.1007/s11627-014-9611-7

[B50] Yuan F. Leng B. Wang B. (2016a). Progress in Studying Salt Secretion from the Salt Glands in Recretohalophytes: How Do Plants Secrete Salt? *Front. Plant Sci.* 7:977. 10.3389/fpls.2016.00977 27446195 PMC4927796

[B51] Yuan F. Lyu M.-J. A. Leng B.-Y. Zhu X.-G. Wang B.-S. (2016b). The transcriptome of NaCl-treated Limonium bicolor leaves reveals the genes controlling salt secretion of salt gland. *Plant Mole. Biol.* 91 241–256. 10.1007/s11103-016-0460-0 26936070

[B52] Yuan F. Leng B. Zhang H. Wang X. Han G. Wang B. (2019). A WD40-Repeat Protein From the Recretohalophyte Limonium bicolor Enhances Trichome Formation and Salt Tolerance in Arabidopsis. *Front. Plant Sci.* 10:1456. 10.3389/fpls.2019.01456 31781150 PMC6861380

[B53] Yuan F. Liang X. Li Y. Yin S. Wang B. (2018). Methyl jasmonate improves tolerance to high salt stress in the recretohalophyte Limonium bicolor. *Funct. Plant Biol.* 46 82–92. 10.1071/FP18120 30939260

[B54] Yuan F. Lyu M. J. Leng B. Y. Zheng G. Y. Feng Z. T. Li P. H. (2015). Comparative transcriptome analysis of developmental stages of the Limonium bicolor leaf generates insights into salt gland differentiation. *Plant Cell Environ.* 38 1637–1657. 10.1111/pce.12514 25651944

