# Supplementary material for: Coupled Development of Salt Glands, Stomata, and Pavement Cells in Limonium bicolor
Source: Front Plant Sci. 2021 Dec 9;12:745422. doi: 10.3389/fpls.2021.745422 (PMC8695552; doi:10.3389/fpls.2021.745422)
Supplement: Supplementary file 1 [file Table_1.DOCX]

Table S1 Correlation analysis between total salt glands (Total_SG) and the other four parameters: total stomata (Total_ST), total pavement cells (Total_PC), leaf area (Leaf_area) and pavement cell area (PC_Area) upon salicylic acid treatment using Pearson’s correlation analysis.

| **Correlations** | | | | | | | |
| --- | --- | --- | --- | --- | --- | --- | --- |
| **SA** | **Mean** | **Std. D** | Total_SG | Total_ST | Total_PC | Leaf_Area | PC_Area |
| Total_SG | 2.675E+02 | 5.695E+01 | 1.000 |  |  |  |  |
| Total_ST | 1.289E+03 | 4.626E+02 | 0.753^**^ | 1.000 |  |  |  |
| Total_PC | 1.069E+04 | 1.978E+03 | 0.639^**^ | 0.636^**^ | 1.000 |  |  |
| Leaf_Area | 2.006E+01 | 2.359E+00 | 0.415^**^ | 0.491^**^ | 0.219^*^ | 1.000 |  |
| PC_Area | 1.408E-03 | 4.051E-04 | 0.715^**^ | 0.609^**^ | 0.511^**^ | 0.256^*^ | 1.000 |
| **. Correlation is significant at the 0.01 level (2-tailed). | | | | | | | |
| *. Correlation is significant at the 0.05 level (2-tailed). | | | | | | | |
