# Supplementary material for: Coupled Development of Salt Glands, Stomata, and Pavement Cells in Limonium bicolor
Source: Front Plant Sci. 2021 Dec 9;12:745422. doi: 10.3389/fpls.2021.745422 (PMC8695552; doi:10.3389/fpls.2021.745422)
Supplement: Supplementary file 2 [file Table_2.DOCX]

Table S2 Correlation analysis between total salt glands (Total_SG) and the other four parameters upon brassinolide treatment using Pearson’s correlation analysis.

| **Correlations** | | | | | | | |
| --- | --- | --- | --- | --- | --- | --- | --- |
| **BR** | **Mean** | **Std. D** | Total_SG | Total_ST | Total_PC | Leaf_Area | PC_Area |
| Total_SG | 1.925E+02 | 5.886E+01 | 1.000 |  |  |  |  |
| Total_ST | 1.218E+03 | 6.096E+02 | 0.791^**^ | 1.000 |  |  |  |
| Total_PC | 7.390E+03 | 2.922E+03 | 0.778^**^ | 0.883^**^ | 1.000 |  |  |
| Leaf_Area | 1.238E+01 | 6.051E+00 | 0.857^**^ | 0.885^**^ | 0.884^**^ | 1.000 |  |
| PC_Area | 1.716E-03 | 4.168E-04 | 0.497^**^ | 0.590^**^ | 0.591^**^ | 0.584^**^ | 1.000 |
| **. Correlation is significant at the 0.01 level (2-tailed). | | | | | | | |
