# Supplementary material for: Coupled Development of Salt Glands, Stomata, and Pavement Cells in Limonium bicolor
Source: Front Plant Sci. 2021 Dec 9;12:745422. doi: 10.3389/fpls.2021.745422 (PMC8695552; doi:10.3389/fpls.2021.745422)
Supplement: Supplementary file 3 [file Table_3.DOCX]

Table S3 Correlation analysis between total salt glands (Total_SG) and the other four parameters upon methyl jasmonate treatment using Pearson’s correlation analysis.

| **Correlations** | | | | | | | |
| --- | --- | --- | --- | --- | --- | --- | --- |
| **MeJA** | **Mean** | **Std. D** | Total_SG | Total_ST | Total_PC | Leaf_Area | PC_Area |
| Total_SG | 2.573E+02 | 7.277E+01 | 1 |  |  |  |  |
| Total_ST | 1.757E+03 | 3.415E+02 | 0.486^**^ | 1 |  |  |  |
| Total_PC | 1.031E+04 | 2.211E+03 | 0.467^**^ | 0.439^**^ | 1 |  |  |
| Leaf_Area | 1.760E+01 | 5.388E+00 | 0.761^**^ | 0.407^**^ | 0.344^*^ | 1 |  |
| PC_Area | 1.638E-03 | 3.167E-04 | 0.167 | 0.309^*^ | 0.246 | 0.049 | 1 |
| **. Correlation is significant at the 0.01 level (2-tailed). | | | | | | | |
| *. Correlation is significant at the 0.05 level (2-tailed). | | | | | | | |
