# Supplementary material for: Coupled Development of Salt Glands, Stomata, and Pavement Cells in Limonium bicolor
Source: Front Plant Sci. 2021 Dec 9;12:745422. doi: 10.3389/fpls.2021.745422 (PMC8695552; doi:10.3389/fpls.2021.745422)
Supplement: Supplementary file 4 [file Table_4.DOCX]

Table S4 Correlation analysis between total salt glands (Total_SG) and the other four parameters upon gibberellic acid treatment using Pearson’s correlation analysis.

| **Correlations** | | | | | | | |
| --- | --- | --- | --- | --- | --- | --- | --- |
| **GA_3_** | **Mean** | **Std. D** | Total_SG | Total_ST | Total_PC | Leaf_Area | PC_Area |
| Total_SG | 1.853E+02 | 4.969E+01 | 1.000 |  |  |  |  |
| Total_ST | 1.218E+03 | 3.016E+02 | 0.619^**^ | 1.000 |  |  |  |
| Total_PC | 7.345E+03 | 1.992E+03 | 0.568^**^ | 0.633^**^ | 1.000 |  |  |
| Leaf_Area | 1.231E+01 | 3.322E+00 | 0.597^**^ | 0.690^**^ | 0.632^**^ | 1.000 |  |
| PC_Area | 1.522E-03 | 4.399E-04 | 0.353^**^ | 0.401^**^ | 0.420^**^ | 0.394^**^ | 1.000 |
| **. Correlation is significant at the 0.01 level (2-tailed). | | | | | | | |
