# Supplementary material for: Coupled Development of Salt Glands, Stomata, and Pavement Cells in Limonium bicolor
Source: Front Plant Sci. 2021 Dec 9;12:745422. doi: 10.3389/fpls.2021.745422 (PMC8695552; doi:10.3389/fpls.2021.745422)
Supplement: Supplementary file 5 [file Table_5.DOCX]

Table S5 Correlation analysis between total salt glands (Total_SG) and the other four parameters upon abscisic acid treatment using Pearson’s correlation analysis.

| **Correlations** | | | | | | | |  |
| --- | --- | --- | --- | --- | --- | --- | --- | --- |
| **ABA** | **Mean** | **Std. D** | Total_SG | Total_ST | Total_PC | Leaf_Area | PC_Area | |
| Total_SG | 2.316E+02 | 4.386E+01 | 1.000 |  |  |  |  | |
| Total_ST | 1.382E+03 | 4.826E+02 | 0.428^**^ | 1.000 |  |  |  | |
| Total_PC | 8.934E+03 | 2.040E+03 | 0.420^**^ | 0.662^**^ | 1.000 |  |  | |
| Leaf_Area | 1.384E+01 | 2.849E+00 | 0.593^**^ | 0.682^**^ | 0.690^**^ | 1.000 |  | |
| PC_Area | 1.078E-03 | 2.792E-04 | 0.474^**^ | 0.541^**^ | 0.500^**^ | 0.424^**^ | 1.000 | |
| **. Correlation is significant at the 0.01 level (2-tailed). | | | | | | | | |
