# Supplementary figures and images for: Coupled Development of Salt Glands, Stomata, and Pavement Cells in Limonium bicolor
Source: Front Plant Sci. 2021 Dec 9;12:745422. doi: 10.3389/fpls.2021.745422 (PMC8695552; doi:10.3389/fpls.2021.745422)

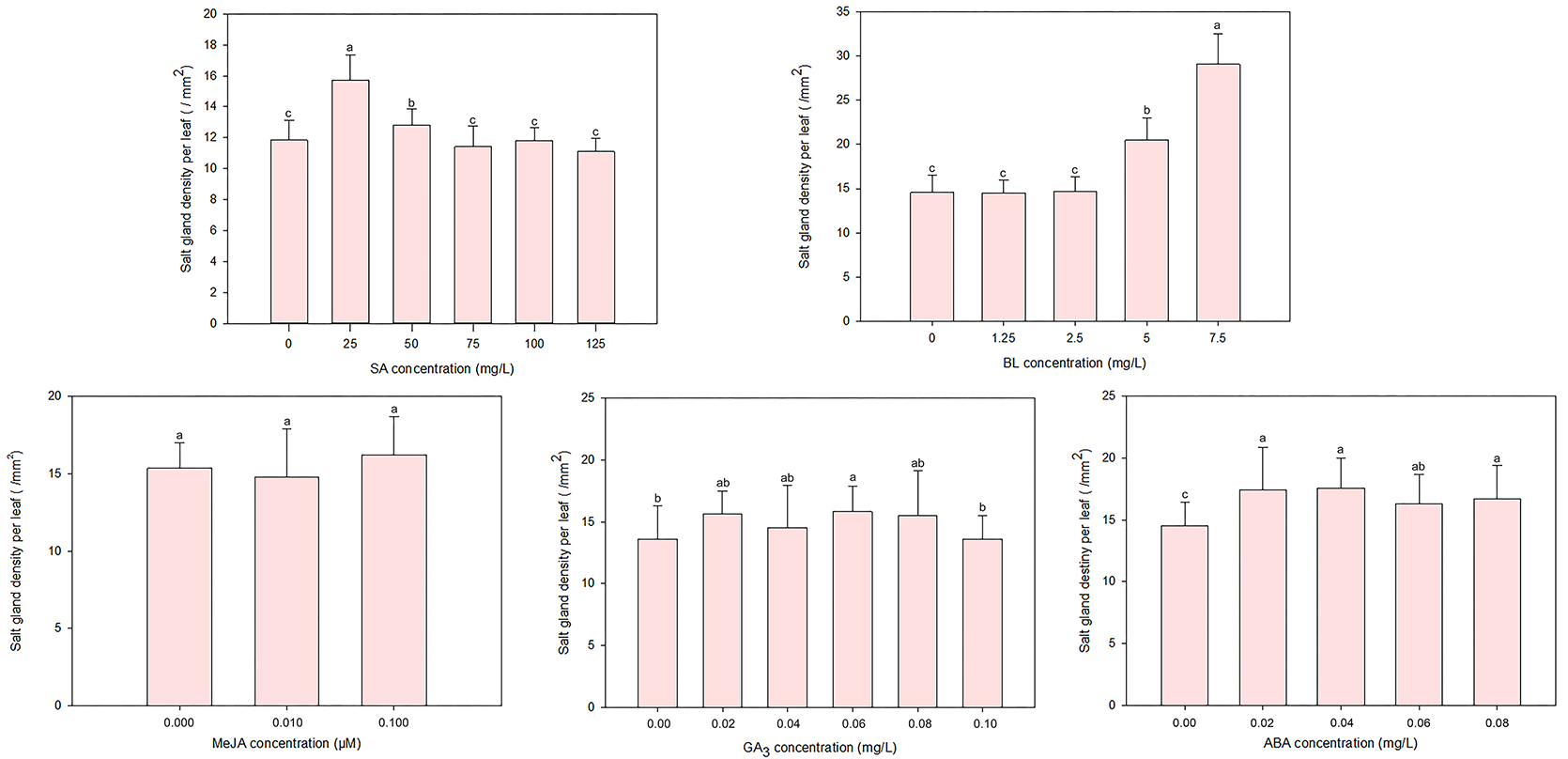

Supplement: Supplementary Figure 1 — Salt gland density of the first true leaf under different hormone treatments. [file Image_1.TIF]
